# Endothelial Caspase-8 prevents fatal necroptotic hemorrhage caused by commensal bacteria

**DOI:** 10.1038/s41418-022-01042-8

**Published:** 2022-07-23

**Authors:** Stefanie M. Bader, Simon P. Preston, Katie Saliba, Adam Lipszyc, Zoe L. Grant, Liana Mackiewicz, Andrew Baldi, Anne Hempel, Michelle P. Clark, Thanushi Peiris, William Clow, Jan Bjelic, Michael D. Stutz, Philip Arandjelovic, Jack Teale, Fashuo Du, Leigh Coultas, James M. Murphy, Cody C. Allison, Marc Pellegrini, Andre L. Samson

**Affiliations:** 1 The Walter and Eliza Hall Institute of Medical Research, Parkville, VIC Australia; 2 Department of Medical Biology, University of Melbourne, Parkville, VIC Australia; 3 Gladstone Institutes, San Francisco, CA USA

**Keywords:** Experimental models of disease, Cell biology, Microbiology, Inflammation

## Abstract

Caspase-8 transduces signals from death receptor ligands, such as tumor necrosis factor, to drive potent responses including inflammation, cell proliferation or cell death. This is a developmentally essential function because in utero deletion of endothelial Caspase-8 causes systemic circulatory collapse during embryogenesis. Whether endothelial Caspase-8 is also required for cardiovascular patency during adulthood was unknown. To address this question, we used an inducible Cre recombinase system to delete endothelial *Casp8* in 6-week-old conditionally gene-targeted mice. Extensive whole body vascular gene targeting was confirmed, yet the dominant phenotype was fatal hemorrhagic lesions exclusively within the small intestine. The emergence of these intestinal lesions was not a maladaptive immune response to endothelial Caspase-8-deficiency, but instead relied upon aberrant Toll-like receptor sensing of microbial commensals and tumor necrosis factor receptor signaling. This lethal phenotype was prevented in compound mutant mice that lacked the necroptotic cell death effector, MLKL. Thus, distinct from its systemic role during embryogenesis, our data show that dysregulated microbial- and death receptor-signaling uniquely culminate in the adult mouse small intestine to unleash MLKL-dependent necroptotic hemorrhage after loss of endothelial Caspase-8. These data support a critical role for Caspase-8 in preserving gut vascular integrity in the face of microbial commensals.

## Introduction

Programmed cell death is essential for the organized removal of diseased and dysfunctional cells. Inflammatory ligands, such as tumor necrosis factor (TNF), are key inducers of programmed cell death when they bind their cognate receptors [1–3]. Paradoxically, these same ligands can promote cell proliferation and survival, depending on the relative stoichiometry of intracellular adaptor and effector molecules [4–7]. For example, while TNF-induced RIPK1 signaling can promote pro-survival NF-kB signaling [4–6], it can alternatively trigger cell death when the activities of pro-survival factors such as cFLIP or cIAP are insufficient [8–10]. More specifically, TNF-induced RIPK1 signaling can trigger Caspase-8-dependent apoptosis [11], or in the absence of Caspase-8 activity can associate with RIPK3 and MLKL to cause necroptosis [12–15]–a lytic form of cell death implicated in the pathogenesis of numerous inflammatory disorders including Crohn’s disease and ulcerative colitis [16–19]. Gene-targeted mice lacking Caspase-8 in all tissues, and mice with endothelial cell-specific deletion of *Casp8*, die in utero due to the aberrant induction of necroptosis [20–23]. This is partially ameliorated by the loss of TNF receptors indicating that while TNF contributes to disease, other pro-death ligands such as Toll-like receptor (TLR) agonists may also promote this lethal phenotype [24]. Intriguingly, when *Casp8* deletion was induced in the endothelium post-partum, mice were relatively healthy but displayed necroptosis-independent impaired retinal angiogenesis [25]. Here, we sought to clarify and determine the role of Caspase-8 in the homeostasis of endothelial cells in adult mice. We interbred *Casp-8* floxed mice with mice expressing a tamoxifen inducible Cre under the control of the endothelial-specific *Cdh5* promoter to generate *Casp8-Cdh5-CreERT2* (referred to as C8-endo). Following tamoxifen dosing, C8-endo mice succumbed to extensive gastrointestinal hemorrhage. Despite equivalent gene targeting throughout the mice, hemorrhage was localised to the small intestine, indicating that this anatomical site was susceptible and responsible for lethality. Histologically, the phenotype is characterised by progressive destruction of capillaries within small intestinal villi. The entire phenotype caused by deletion of endothelial Caspase-8 was prevented in mice that lacked the necroptotic effector protein MLKL. Endogenous TNF- and TLR-signaling were also key drivers of pathology, suggesting that necroptosis triggered by these initiators was responsible for small intestinal hemorrhage in C8-endo mice. Our data show that Caspase-8 is indispensable for the maintenance of vascular integrity in the small bowel, but is redundant in the homeostasis of other endothelial cell niches.

## Results

### C8-endo mice succumb to catastrophic gastrointestinal hemorrhage

**Fig. 1:**
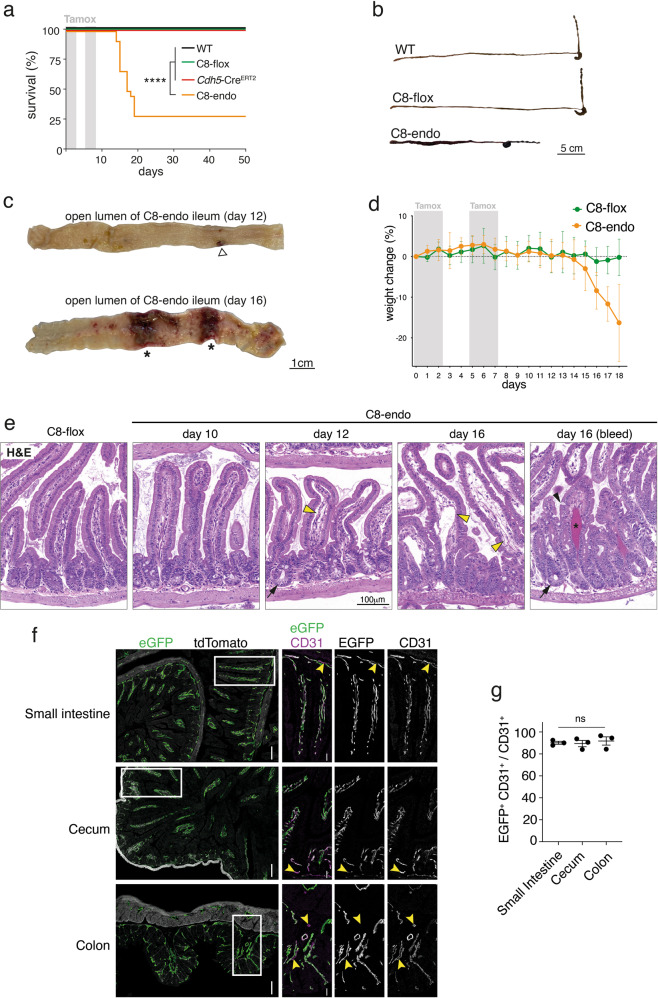
C8-endo mice succumb to small intestinal hemorrhage. **a** The proportion of mice that became moribund and required euthanasia. Gray boxes indicate days of tamoxifen administration. Data are combined from five independent experiments (*n* = 23–27 mice). ****P < 0.0001 via Log-rank Mantel–cox test. **b** Representative images of the gastrointestinal tract from a moribund C8-endo mouse and matched C8-flox and wild-type mice. **c** Representative images of the luminal face of the ileum from C8-endo mice 12 or 16 days after commencing tamoxifen treatment. Open arrow indicates a nascent hemorrhagic lesion. Asterisks indicate larger more developed hemorrhagic lesions. **d** Relative changes to the body weight of mice. Mean ± s.d. from *n* = 5 mice per genotype are shown. Gray boxes indicate days of tamoxifen administration. **e** Representative H&E-stained sections of the small intestines of control (C8-flox) mice and C8-endo mice 10, 12 and 16 days post-tamoxifen. Yellow arrowheads indicate compromised blood vessels. Asterisk indicates hemorrhagic transformation. Arrows indicate empty crypts. Black arrowhead indicates a Goblet cell. **f** Immunofluorescence images of sections from C8-endo-ROSA mice five days after the first dose of tamoxifen. Right panels are enlarged micrographs of the inset white boxes in the left-most panels. Scale bars in left panels indicate 100μM. Scale bars in right panels indicate 30 μM. Arrowheads indicate GFP-negative endothelial cells. Micrographs are representative of at least six specimens per group. **g** Quantification of the percentage of EGFP-CD31-positive cells over the total number of CD31-positive cells across the small and large intestine of C8-endo mice. Data are the mean ± s.e.m. of >100 cells/group/mouse. Symbols represent individual C8-endo mice. ns via one-way ANOVA with Geisser-Greenhouse correction.

Tamoxifen was administered to delete *Casp8* from the endothelium of six week old C8-endo mice. Two-to-three weeks after commencing tamoxifen treatment, approximately 75% of C8-endo mice were euthanized because of illness (Fig. 1a). Necropsy revealed extensive hemorrhage and shortening of the small intestine (Fig. 1b). Remarkably, this pathology was restricted to the small bowel, with the remaining gastrointestinal tract and other organs being macroscopically normal and devoid of hemorrhage. The same hemorrhagic phenotype manifested when tamoxifen was administered via oral or intraperitoneal routes. Longitudinal study of C8-endo mice showed that small hemorrhagic lesions appeared in the small bowel approximately 12 days after tamoxifen treatment (open triangle; Fig. 1c). These lesions progressively increased in size and number, eventually affecting a large proportion of the small bowel (from the proximal duodenum to the terminal ileum; asterisks; Fig. 1c). The progression of gut hemorrhage in C8-endo mice was closely mirrored by weight loss (Fig. 1d). Histological changes to the small intestine in C8-endo mice also emerged from ~12 days post-tamoxifen. In particular, the capillary network of villi became increasingly disorganized and detached from the overlying epithelium (open arrowheads; Fig. 1e) leading to hemorrhage (asterisk; Fig. 1e). These primary vascular changes were closely followed by crypt emptying and elongation of crypts and villi (closed arrows; Fig. 1e). No histological differences were noted in the stomach, cecum, colon or in extra-intestinal tissues such as the kidney between C8-endo mice and control C8-flox mice (*Cdh5*-Cre^ERT2^ *Casp8*^fl/fl^ mice that had not received tamoxifen and thus maintained normal endothelial Caspase-8 expression; Fig. S1A). Thus, loss of endothelial Caspase-8 in adult mice causes progressively worsening hemorrhage only in the small bowel.

The sparing of other organs from hemorrhage led us to speculate that our gene-targeting strategy was not uniformly deleting Caspase-8 from all endothelial beds. To address this possibility, we crossed *ROSA dTomato-EGFP* reporter mice [26] with C8-endo mice to confirm EGFP expression and thus Cre activity across the endothelium (as a surrogate for *Casp8* deletion efficiency). Extensive EGFP expression was observed throughout the endothelium in all tested organs including the small intestine, cecum and colon (Fig. 1f and Fig. S1B). No expression of EGFP was observed in CD31-negative cells (Fig. 1f), with quantification showing that ~90% of CD31-positive endothelial cells across the small and large intestines co-expressed EGFP. Thus, specific, efficient, and uniform gene-targeting had been achieved across all vascular beds in C8-endo mice (Fig. 1g). These results suggest that fatal gut hemorrhage in C8-endo mice was not due to non-uniform or non-specific Cre activity, but rather indicative of a protective role for endothelial Caspase-8 that uniquely, or preferentially, operates in the small bowel.

To determine whether loss of endothelial Caspase-8 can have extra-intestinal consequences, we infected C8-endo mice with lymphocytic choriomeningitis virus (LCMV)–a virus with endothelial tropism that causes systemic infections especially in Caspase-8-deficient mice [27]. As expected, C8-endo mice displayed altered responses to LCMV-infection, with exaggerated non-hemorrhagic edema and leukocyte infiltration in the skin and peritoneum (arrows; Fig. S1C). Collectively, these data confirm that despite its widespread deletion, the vital role of endothelial Caspase-8 is to constitutively guard against small intestinal hemorrhage.

### Hemorrhage in C8-endo mice is due to unrestrained necroptosis

**Fig. 2:**
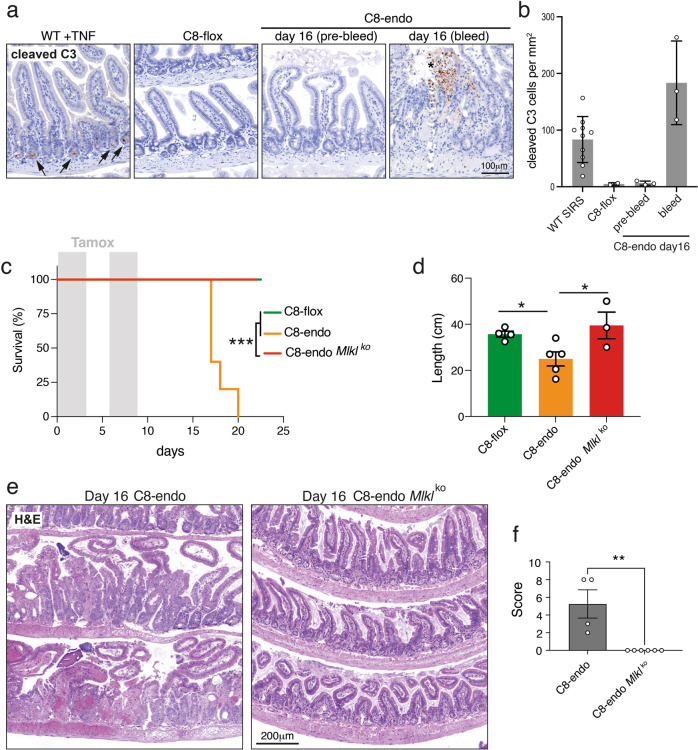
MLKL-dependent necroptosis is driving hemorrhage in C8-endo mice. **a** Micrographs (**a**) and quantitation (**b**) of cleaved Caspase-3 staining in the small bowel of a wild-type mouse 15 hours after TNF administration (WT + TNF), a control mouse (C8-flox) and a C8-endo mouse 16 days after starting tamoxifen treatment. Micrographs show the remodeled intestinal wall before (pre-bleed) and after hemorrhage (bleed) in C8-endo mice. Arrows indicate cleaved Caspase-3 in crypts. Asterisk indicates cleaved Caspase-3 within a hemorrhage. **b** Data are mean ± s.d. from *n* = 10 for WT + TNF; *n* = 2 for C8-flox and *n* = 3 for C8-endo. Over 100 villi were analyzed per mouse. Symbols represent individual mice. **c** The proportion of mice that became moribund and required euthanasia. Gray boxes indicate days of tamoxifen administration. Data are from one cohort (*n* = 4–5 mice per group) and representative of 3 independent experiments. ***p < 0.001 via Log-rank Mantel–cox test. **d** Lengths of the small intestine from matched control (C8-flox) mice, C8-endo-*Mlkl*^*ko*^ mice and moribund C8-endo mice (same cohort as in **c**). Data are mean ± s.e.m. *p < 0.05 via one-way ANOVA with Holm-Sidak’s multiple comparison test. Symbols represent individual mice. **e** H&E-stained sections of mice 16 days after starting tamoxifen treatment. **f** Histopathological scores of H&E-stained sections of the small intestine from moribund C8-endo and matched C8-endo-*Mlkl*^*ko*^ mice. Data are mean ± s.e.m. **p < 0.01 via unpaired two-sided t-test. Symbols represent individual mice.

As Caspase-8 is a master regulator of multiple cell death programs [28, 29], we next investigated whether dysregulated cell death was responsible for the phenotype of C8-endo mice. While elevated apoptosis was observed in the small intestines of TNF-treated wild-type mice (arrows; Fig. 2a, b), apoptosis was not increased in the small intestines of C8-endo mice prior to hemorrhage (asterisk; Fig. 2a, b). Rather, lethality was completely prevented in C8-endo mice which lacked MLKL–the terminal effector of the necroptotic cell death pathway [14, 15] (Fig. 2c). These data align with the notion that Caspase-8 activity triggers apoptosis [10], whereas its absence licenses MLKL-dependent necroptotic cell death [22, 23, 30]. Indeed, compound loss of MLKL completely prevented inflammatory shortening of the small bowel (Fig. 2d) and histological remodeling of intestinal capillaries, crypts and villi in C8-endo mice (Fig. 2e, f). These data strongly suggest that loss of endothelial Caspase-8 causes hemorrhage, not by triggering apoptosis, but instead by eliciting MLKL-dependent necroptosis in the small intestine.

The mechanistic basis for necroptosis in the C8-endo model differs from that caused by deletion of epithelial Caspase-8. Whereas necroptosis due to epithelial Caspase-8 deletion involves increased RIPK3 and MLKL expression and Goblet cell loss in the small intestine [19, 31, 32], no alterations in the levels of RIPK3, MLKL or Goblet cells were noted in C8-endo mice (Fig. S2A–C). Despite considerable efforts, we were unable to detect phosphorylated MLKL via Western blot in the small intestines of C8-endo mice. This is likely a technical caveat due to the modest specificity of existing anti-mouse phospho-MLKL antibodies [33]. Altogether, these points of difference, together with strong MLKL expression in the capillaries of the small intestine (arrowheads; Fig. S2B), led us to propose that low levels of progressive endothelial necroptosis are responsible for fatal small intestinal hemorrhage in C8-endo mice.

### Aberrant TNF- and microbial-signaling cause necroptotic hemorrhage

**Fig. 3:**
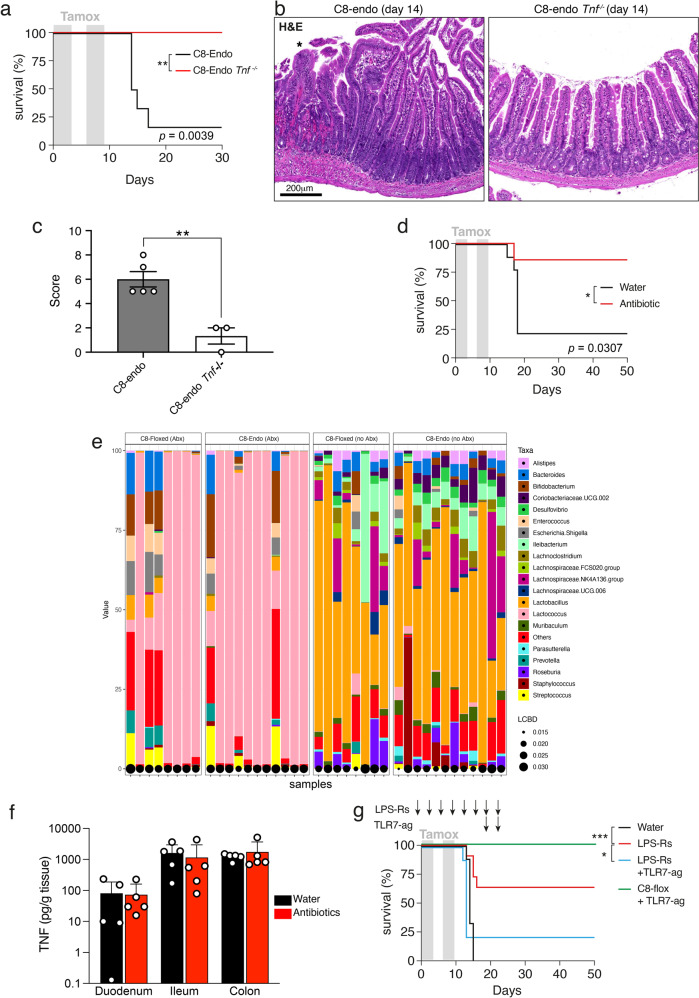
Necroptotic hemorrhage in C8-endo mice is driven by TNF and commensal bacteria. **a** The proportion of mice that became moribund and required euthanasia. Gray boxes indicate days of tamoxifen administration. Data are from one cohort (*n* = 6 mice per group) and representative of 2 independent experiments. **p < 0.01 via Log-rank Mantel–cox test. **b** H&E-stained sections of mice 14 days after starting tamoxifen treatment. **c** Histopathological scores of H&E-stained sections of the small intestine from moribund C8-endo and matched C8-endo *Tnf*^*−/−*^ mice. Data are mean ± s.e.m. **p < 0.01 via unpaired two-sided *t*-test. Symbols represent individual mice. **d** The proportion of mice that became moribund and required euthanasia. Gray boxes indicate days of tamoxifen administration. C8-endo mice drank vehicle (water) or an antibiotic cocktail starting one week prior to tamoxifen treatment. Data are from one cohort (*n* = 7–9 per group). *p < 0.05 via Log-rank Mantel–cox test. **e** The relative abundance of bact**e**rial taxonomic families (Taxa) in samples from the indicated mice treated with an antibiotic cocktail (Abx) or without antibiotics (no Abx). Data are from one experimental cohort. The local contribution to beta diversity (LCBD) is shown. **f** Mice were sacrificed 12 days following the first dose of tamoxifen and TNF levels quantitated from the indicated tissues by ELISA. Data are mean ± s.e.m. Symbols represent individual mice. **g** The proportion of mice that became moribund and required euthanasia. Gray boxes indicate days of tamoxifen administration. Arrows indicate timing of LPS-Rs or TLR7-Ag administration. Data are combined from two cohorts (*n* = 5–11 mice per group). *p < 0.05 and ***p < 0.001 via Log-rank Mantel–cox test.

We next sought to determine which inflammatory ligands induce necroptosis in C8-endo mice. As TNF is a well-characterized initiator of necroptotic signaling [34], we administered TNF-neutralizing antibodies one day prior to tamoxifen treatment and every third day thereafter. TNF neutralization prevented pathology in almost all C8-endo mice (Fig. S3A). Furthermore, akin to C8-endo-*Mlkl*^*ko*^ mice, compound mutant C8-endo mice that lacked TNF showed no disease symptoms (Fig. 3a) and no pathological remodeling of the small intestine (Fig. 3b, c). Thus, like MLKL, TNF is critical to the etiology of hemorrhagic lesions following loss of endothelial Caspase-8. The TNF-dependency of the C8-endo model is in stark contrast to models where necroptosis is instead triggered by epithelial Caspase-8 deletion [35].

To investigate the source of TNF, we reconstituted C8-floxed mice with bone marrow from wild-type or TNF-deficient donors, then treated chimeric mice with tamoxifen to delete endothelial Caspase-8. All C8-endo animals that received wild-type bone marrow succumbed to gastrointestinal hemorrhage, however, mice receiving TNF-deficient bone marrow showed modest amelioration of disease (Fig. S3B). These findings denote that hematopoietic-derived TNF partially contributes to lethal necroptosis. To define which immune cells mediate the phenotype of C8-endo mice, we depleted specific cell populations prior to tamoxifen treatment. Macrophages, T- and B-cells were depleted with anti-F4/80, anti-CD3 or anti-CD79b antibodies. Despite confirming specific cell depletion in the lamina propria of C8-endo mice (Fig. S3C), necroptotic hemorrhage was neither prevented nor delayed (Fig. S3D). Similarly, to investigate a role for adaptive immunity, C8-endo chimeras were generated from wild-type or *Rag1*-deficient bone marrow donors, however, all recipient mice succumbed with the same kinetics after tamoxifen treatment (Fig. S3E). Thus, despite requiring multiple sources of TNF, the necroptotic phenotype of C8-endo mice is not a maladaptive immune response and does not rely upon one single population of macrophages, T- and B- cells.

Besides TNF, lipopolysaccharide (LPS) and other pathogen-associated molecular patterns (PAMPs) are potent initiators of necroptotic signaling [34, 36]. Thus, we speculated that the focusing of pathology to the gut may be due to microbial signals. To address this, mice were pre-treated with an antibiotic cocktail for one week prior to tamoxifen treatment and then maintained on antibiotics throughout the experiment. This treatment regime markedly reduced the diversity of commensal bacteria (Fig. S3F) and significantly reduced mortality in C8-endo mice (Fig. 3d). Given that our C8-endo mice were housed under specific pathogen-free conditions, these data indicated that non-pathogenic commensal microbiota induced necroptotic hemorrhage. Moreover, as use of a single antibiotic failed to prevent hemorrhage in C8-endo mice, an array of commensal bacteria likely trigger disease. Although closer examination of the microbiome did not highlight overt differences in the microbiota of control versus C8-endo mice (Fig. 3e), the identity of necroptosis-induced bacteria in C8-endo mice remain of outstanding interest. Notably, as treatment with the antibiotic cocktail did not alter TNF levels in the small intestine, the protection offered by antibiotics was not due to abrogated TNF signaling (Fig. 3f). Thus, dysregulated TNF- and microbial-sensing are both essential, but neither are sufficient, to cause small intestinal hemorrhage after loss of endothelial Caspase-8.

To confirm the role of dysregulated microbial sensing, LPS from *Rhodobacter spheroides* (LPS-Rs) was administered to C8-endo mice. LPS-Rs is an inhibitor of endogenous TLR4 signaling [37, 38]. As shown in Fig. 3g, LPS-Rs treatment significantly protected against fatal gastrointestinal hemorrhage. Thus, endogenous TLR4 signaling, presumably via LPS from commensal microbiota, promoted necroptotic hemorrhage in C8-endo mice. Furthermore, to determine whether non-bacterial TLR agonists could promote hemorrhagic necroptosis, C8-endo mice were administered the potent TLR7 agonist, Gardiquimod (TLR7-ag) [39] along with LPS-Rs. Such TLR7 activation rapidly induced gastrointestinal hemorrhage and illness in mice, confirming that activation of other TLRs could drive pathology independently of TLR4-signaling (Fig. 3g).

Collectively, we have defined that endothelial caspase-8 is needed in adult mice to protect against severe TNF- and microbial-TLR-driven necroptotic hemorrhage. This role uniquely manifests in the small intestine, and mechanistically differs from the systemic role of Caspase-8 during embryogenesis [20–23], from the post-natal role of Caspase-8 in retinal angiogenesis [25], and from the role of Caspase-8 in the epithelium of the small intestine [19, 32, 35].

## Discussion

Our studies demonstrated that Caspase-8 is critically required to protect gut endothelial cells from microbiota-triggered necroptosis. However, importantly, in the absence of abundant microbial products, Caspase-8 was dispensable for endothelial cell hemostasis across all vascular beds. We found that after loss of endothelial Caspase-8, commensal bacteria can initiate necroptosis throughout the small intestine, but the cecum and large intestine were not susceptible to this phenotype despite abundant microorganisms in these regions [40, 41]. One possibility for this regionality is that undefined features of the large intestine, for example diminished PAMP-signaling, increases its tolerance to higher microbial loads. We speculate that such features may be less prevalent in the small intestine; a section of the gut that does not accommodate very high microbial loads and thus appears more susceptible to microbial-induced necroptosis when endothelial Caspase-8 levels are compromised. Heightened sensitivity of the small intestine to translocated microbial products may be a key surveillance mechanism to facilitate robust responses to enteric pathogens. Indeed, several bacterial pathogens actively inhibit necroptosis, at least in intestinal epithelial cells, perhaps to prevent their host from dying and hence facilitating shedding and transmission [42, 43]. Some studies suggest that enteropathogenic *Escherichia coli* have the capacity to also interact with endothelial cells, not just epithelial cells, to facilitate disease pathogenesis and spread [44].

We showed that disease manifestation in C8-endo mice was not immediate. This may be due to the kinetics of *Casp8* deletion or, more likely, due to the time required for PAMPs to translocate to TLRs, promote TNF production and induce necroptosis in a positive feedback loop. Although the hemopoietic compartment exacerbates the phenotype, it is not essential for disease in C8-endo mice. It is intriguing to speculate that some commensal organisms may be more prone to inducing disease in Caspase-8-insufficent endothelium, whilst others may produce forms of LPS that are anti-inflammatory in much the same way as LPS-Rs. Our C8-endo mice may serve as a powerful tool for identifying microbial communities that are more conducive to causing/ameliorating inflammatory disease. A plethora of studies have revealed a striking bacterial dysbiosis preceding the development of inflammatory bowel disease (IBD) and during established illness [45–47]. Furthermore, studies have demonstrated that models of Caspase-8 deficiency produce IBD-like manifestations [18, 19, 31, 32], and that mutations in the Caspase-8 gene are a monogenic cause of very early onset IBD [48].

Defective epithelial and/or immune cells, rather than dysfunctional endothelium, are more commonly associated with IBD. However, as resident cell types within the gastrointestinal wall cooperatively maintain gut shape and function [49], it stands to reason that endothelial defects also precipitate inflammatory gut disease. Indeed, upon completion of our study, another group has separately reported that loss of endothelial Caspase-8 in adult mice precipitates small intestinal hemorrhage [50]. Our findings largely overlap or extend the observations of Tisch and colleagues [50]. For instance, while polymicrobial products broadly trigger hemorrhage in C8-endo mice, we find that endogenous TLR4-signaling is especially critical for this necroptotic phenotype. One notable difference, however, is that we observed no obvious correlation between the sites of hemorrhagic lesioning and Peyer’s patches; consistent with hemorrhage being independent of adaptive or innate immune cells and independent of hematopoietic-derived TNF. Irrespective, these studies elucidate a critical role for Caspase-8 in maintaining the integrity of the gastrointestinal endothelium under steady-state conditions by preventing catastrophic inflammation and hemorrhage. Even minor aberrations in this pathway could have dramatic clinical consequences for gastrointestinal health.

## Methods

### Materials

Antibodies used for Western blotting were 1 μg/mL of rat anti-mouse MLKL (clone 5A6; produced in-house; [33] available from Millipore as MABC1634), 1 μg/mL rat anti-mouse RIPK3 (clone 8G7; produced in-house; [51] available from Millipore as MABC1595), 1:1000 dilution of rabbit anti-Caspase-8 (clone D35G2; Cell Signaling Technology #4790) and 1:2000 dilution of mouse anti-GAPDH (Millipore MAB374). Secondary antibodies for immunoblotting were: horseradish peroxidase (HRP)-conjugated goat anti-rat IgG (Southern Biotech 3010-05), HRP-conjugated goat anti-mouse IgG (Southern Biotech 1010-05), and HRP-conjugated goat anti-rabbit IgG (Southern Biotech 4010-05). All secondary antibodies for immunoblotting were used at a dilution of 1:10000.

### Mice

*Casp8-Cdh5-CreERT2* mice (C8-endo) were generated by crossing previously published *Casp8*^flox/flox^ mice [27] to those expressing Cre recombinase fused to the estrogen receptor, under the control of the *Cdh5* promoter [52]. Mice were additionally crossed to *Mlkl*^−/−^ animals [53] to generate *Casp8-Cdh5-CreERT2 Mlkl*^−/−^ (C8-endo-Mlkl^ko^). C8-endo mice were separately crossed to *Tnf*^−/−^ mice [54] to generate *Casp8-Cdh5-CreERT2 Tnf*^*−/−*^ (C8-endo *Tnf*^*−/−*^) mice. All C8-endo and compound mutant mice were crossed to a Cre reporter mouse strain to help visualize Cre-mediated gene targeting (*ROSA dTomato-EGFP*) [26]. Cre-ERT2 activity was induced by administering tamoxifen (Sigma) (150 mg/kg) to mice, dissolved in corn oil (Sigma) and delivered by oral gavage as a regimen of three days on, two days off, three days on. *Rag1*^*−/−*^ Ly5.1 mice used in this study have been previously described [55]. Approximately equal numbers of male and female mice were used in this study. When littermates could not be used, mice were randomly allocated to treatment groups after age- and sex-matching by an independent animal technician.

### Chimera generation and antibodies used for in vivo depletion

Six- to ten-week old recipient C8-endo mice were sub-lethally irradiated with two doses of 550 rads (Co-60), spaced four hours apart. Animals were reconstituted via intravenous injection of 5 × 10^6^ bone marrow cells from PBS-flushed femurs and tibias of donor mice. Recipient mice received 200 μg of anti-Thy1.1 (clone 30H12; WEHI Monoclonal Antibody Facility) antibody via intraperitoneal injection to deplete donor and residual recipient T cells. Recipients were maintained on neomycin (1 g/L) in drinking water for ten days. Chimeric animals were bled after twelve weeks to confirm immune reconstitution. For depletion experiments, antibodies targeting TNF (Armenian hamster; 200 mg; clone: TN3-19.12), B cells (CD79b, Armenian hamster; 50 mg; clone: HM79-11) (MyBioSource), T cells (anti-CD3e F(ab’)2; Armenian hamster; 50 mg; clone: 145-2C11), macrophages (F4/80; Rat; 200 mg; clone: CI:A3-1), or isotype controls (Armenian hamster: cat # BE0091 and BE0091-FAB / Rat: cat # BE0090; Bio Xcell) were administered to mice: commencing either one day prior to the first tamoxifen dose and continuing every three days for a total of six doses (anti-TNF, -CD79b and -CD3e), or commencing four days post the first tamoxifen dose, continuing every three days for a total of three doses (anti-F4/80).

### TLR agonists and antibiotic cocktail

Agonists included: LPS (lipopolysaccharide, *E. coli*), LPS-Rs (*Rhodobacter spheroides*) (ultrapure) and Gardiquimod (TLR7-Ag; all Invivogen). C8-endo mice were administered LPS-Rs (1 mg/kg) every two days via oral gavage commencing one day prior to the first tamoxifen dose (Days −1, 1, 3, 5, 7, 9, 11, 13). For combination experiments, TLR7-Ag (5 mg/kg) was administered in addition to LPS-Rs, as two doses following the final dose of tamoxifen (days 11, 13). The following antibiotics were used alone or combined as cocktail dissolved in water: ampicillin (Sigma; 1 g/L), neomycin (Sigma; 1 g/L), metronidazole (Baxter; 1 g/L), enrofloxacin (Baxter; 0.5 g/L), meropenem (Clifford Hallam; 2.5 g/L). Antibiotics were consumed *ad libitum* via drinking water. Antibiotic water was protected from light and changed every two days. Antibiotic administration commenced one week prior to the first dose of tamoxifen and was maintained in drinking water for the duration of the experiment.

### Immune cell isolation and flow cytometry

Intestinal immune cell populations were isolated as described previously [56]. Cells were stained with the following antibodies (all from BD Biosciences) for 30 min at 4 °C: Fc block (anti-CD16/CD32, 1:200) rat anti-CD4 BV421 (clone GK1.5, 1:500), rat anti-CD8 BV510 (clone 53–6.7, 1:500), rat anti-CD19 PerCP-Cy5.5 (clone 1D3, 1:200), rat anti-B220 BV605 (clone RA3-6B2, 1:200), hamster anti-CD3 BV711 (clone 145-2C11, 1:200), rat anti-F4/80 AF488 (clone BM8, 1:200). Stained cells were visualized using a FACSAria Fusion flow cytometer (BD Biosciences). Data was analyzed using FlowJo Software v.10.7 (FlowJo LLC).

### Viral infection

C8-endo mice commenced on antibiotic cocktail therapy via drinking water, followed by tamoxifen as described. Mice were infected one day following with LCMV *Docile* via intravenous tail vein injection of 2 × 10^6^ plaque-forming units under penthrane anesthesia. Animals were euthanized 7-days post-infection.

### TNF-treatment

Seven-week old C57BL/6 J wild-type female mice received 1% hydroxyethylcellulose (Sigma 09368) by oral gavage followed 1 h later by 300 µg/kg recombinant murine TNF (R&D Systems 410-MT-CF) intravenously via tail vein. TNF was dissolved and diluted in endotoxin-free PBS (EMD Millipore TMS-12-A). Core body temperature was recorded by digital rectal probe as baseline before injection and every hour thereafter. Mice with a core body temperature below 30.0 °C were euthanized.

### TNF ELISA

Organs were homogenized in T-PER reagent (ThermoFisher) containing cOmplete mini protease inhibitor cocktail (Sigma) using a TissueLyser II (Qiagen). Homogenates were clarified by centrifugation at 20,000 x g for 10 min at 4 °C. TNF levels were quantified using a mouse TNF ELISA kit according to manufacturer’s instructions (R&D Systems). Absorbance at λ = 450 nm was analyzed on a Hidex chameleon plate reader.

### Western blot

Tissues were lysed in ice-cold RIPA buffer to a concentration of 50 mg/ml (w/v) with a stainless steel bead using a Qiagen TissueLyser II (1 min at 30 Hz). Whole cell/tissue lysates were boiled for 10 min in 1× SDS Laemmli sample buffer (126 mM Tris-HCl, pH 8, 20% v/v glycerol, 4% w/v SDS, 0.02% w/v bromophenol blue, 5% v/v 2-mercaptoethanol), and resolved on 1.5 mm NuPAGE 4–12% Bis-Tris gels (ThermoFisher Scientific NP0335BOX) using MES Running buffer (ThermoFisher Scientific NP000202). After transfer onto polyvinylidene fluoride, membranes were blocked in 5% w/v skim milk powder in TBS-T, probed with primary antibodies (see *Materials* above) then the appropriate HRP-conjugated secondary antibody and signals revealed by enhanced chemiluminescence (Merck P90720) on a ChemiDoc Touch Imaging System (Bio-Rad). Between each probe, membranes were incubated in stripping buffer (200 mM glycine pH 2.9, 1% w/v SDS, 0.5 mM tris(2-carboxyethyl)phosphine) for 30 min at room temperature then re-blocked. Full length uncropped original western blots are provided in Supplementary File 1.

### Histology and immunostaining

Organs were harvested and fixed in 10% neutral buffered formalin for 48 h, followed by ethanol dehydration, paraffin embedding and sectioning. Slides were stained with either hematoxylin and eosin (H&E), or Periodic acid–Schiff (PAS), or immunohistochemically with rabbit anti-mouse cleaved Caspase-3 (Cell Signaling Technology; Product #9661) using the automated Omnis EnVision G2 template (Dako, Glostrup, Denmark). For cleaved Caspase-3 staining, dewaxing was performed with Clearify Clearing Agent (Dako) for 15 min, and antigen retrieval with EnVision FLEX TRS at low pH (Dako) at 97 °C for 30 min. Primary antibodies were diluted 1:300 in EnVision Flex Antibody Diluent (Dako) and incubated at 32 °C for 60 min. HRP-labelled secondary antibodies were applied at 32 °C for 20 min. Slides were counterstained with Mayer hematoxylin, dehydrated, cleared, and mounted with MM24 Mounting Medium (Surgipath-Leica, Buffalo Grove, IL, USA). Stained slides were imaged with an Aperio ScanScope AT (Leica Microsystems, Wetzlar, Germany) or a PANNORAMIC SCAN II (3D HISTECH). For the datasets in Figs. 2f and 3c, a histopathological scoring system (0–3) was used by Phenomics Australia staff, who were blinded to the experimental mouse genotypes, for grading histological changes. The matrix used for histological scoring was the sum of the following parameters: Crypt architecture (0 = normal, 1 = irregular, 2 = moderate crypt loss, 3 = severe crypt loss), Tissue damage (0 = no damage, 1 = discrete lesion, 2 = mucosal erosion, 3 = Extensive mucosal damage/ulceration extending into muscularis or deeper), Inflammatory cell infiltration (0 = occasional infiltration, 1 = increasing leukocyte in lamina propria, 2 = confluence of leukocytes extending into submucosa, 3 = transmural extension of inflammatory infiltrate) and Enterocyte hyperplasia (0 = none, 1 = mild, 2 = moderate, 3 = severe).

### Quantification of cleaved caspase-3 immunostaining

Quantification of cleaved Caspase-3 was performed using a custom macro in Fiji software version 1.53r [57]. Full resolution (x40 magnification) micrographs of a Swiss rolled small intestine sections stained for cleaved Caspase-3 via 3,3’-diaminobenzidine (DAB) and counterstained with hematoxylin were opened in Fiji. One well-oriented region spanning at least 100 villi was chosen per mouse, color deconvolution plugin was then used to separate the DAB signal from the hematoxylin signal. Autothrehold>minimum processing of the isolated hematoxylin signal was performed and converted to a mask. The area of this mask was taken as the “total tissue area”. Thresholding (values 100–255) of the isolated DAB signal was performed, converted to a mask and then subjected to the Analyze Particles plugin (size = 225–3500 pixels^2^; which subjectively approximated the size of apoptotic cells). The number of apoptotic cells was divided by the total tissue area to measure the number of “cleaved Caspase-3-positive cells per mm^2^”.

### Fluorescent imaging and quantification

To visualize dTomato and EGFP expression, tissues were fixed in 4% paraformaldehyde at 4 °C for 120 min, then dehydrated in 30% sucrose at 4 °C overnight. Tissues were frozen in Tissue-Tek Optimal Cutting Temperature (Sakura Finetek) media and cryo-sectioned onto poly-l-lysine glass slides. For CD31 visualization, frozen sections were blocked in normal donkey serum (Jackson Immunoresearch), then stained with rat anti-CD31 (BD Biosciences; Clone MEC 13.3), rabbit anti-GFP (ThermoFisher cat # A11132), donkey anti-rat (AF647) (Jackson cat # 712-605-150), anti-rabbit (DL488) (Jackson cat # 711-485-152) antibodies. Coverslips were mounted using ProLong Gold antifade with DAPI (Molecular Probes). Fluorescent tissue sections were imaged using a Leica TCS SP8 confocal microscope using a 20x/0.75 NA objective and Leica Application Suite software. All image analysis was performed in ImageJ software. Quantification of EGFP expressing endothelium was calculated on smaller regions cropped from maximum projection images. Binary masks of both CD31 and EGFP channels were made by various morphological filters and thresholding signal manually. A mask of the overlap between EGFP and CD31 channels was generated using the Image Calculator feature. This overlap area was measured and presented as a percentage of total CD31 mask area. Where necessary, a despeckle filter was applied to select channels in images displayed in figures for clarity.

To visualize MLKL, intestines were dissected post-mortem, flushed with 10 ml of ice-cold 50% v/v ethanol and 5% v/v acetic acid, Swiss rolled, submerged in ice-cold Milestone freezing medium, and then frozen using PrestoCHILL device (Milstone; settings of 2 min at −40 °C). 8 μm sections were cut on a cryostat and air-dried onto Superfrost slides (ThermoFisher Scientific) for 30 min at room temperature. Sections were fixed for 30 min in ice-cold methanol, then rinsed twice in ice-cold Dulbecco’s PBS and blocked in Tris-balanced salt solution with 0.05% v/v Triton-X100 (TBS-T) supplemented with 10% v/v donkey serum (Sigma-Aldrich D9663) overnight at 4 °C. Sections were incubated overnight at 4 °C in 5 mg/L of rat anti-mouse MLKL antibody (clone 5A6; ref[33].) in TBS-T with 10% v/v donkey serum. Sections were washed three times in TBS-T then incubated overnight at 4 °C in a 1:1000 dilution of AlexaFluor488-conjugated donkey anti-rat IgG (Thermo-Fisher Scientific) supplemented with 0.1 μg/mL Hoechst 33342 (ThermoFisher Scientific H3570) in TBS-T with 10% v/v donkey serum. Sections were washed four times in ice-cold TBS-T, mounted in DAKO fluorescent mounting media (DAKO S3023) and kept in the dark at room temperature until being imaged by 3-dimensional confocal microscopy on an Inverted LSM 880 platform (Zeiss) equipped with the following specifications: 20x/0.8 N.A. PlanApo M27 objective (Zeiss), 405-,488-, 568-, and 640-nm laser lines, and radially-stacked GaASP detectors and ZEN black 2.3 SP1 FP3 v.14.0 capture software.

### Bacterial DNA sequencing

Samples were thawed on ice and DNA was extracted using the DNeasy PowerSoil Pro kit (Qiagen) and the TissueLyser LT (Qiagen) for sample disruption. DNA was normalized and the V4 hypervariable region of the bacterial 16 S rRNA gene was amplified in a PCR reaction (PCR1). Primers included sequences for sequencing adapters and barcodes suitable for the Illumina MiSeq instrument. Amplicons were diluted 1 in 5 and then underwent a second PCR amplification (PCR2) in which dual indexes were attached for downstream sample identification. PCR1 heated samples to 95 °C for 3 min then comprised 20 cycles of 95 °C for 45 s, 50 °C for 60 seconds and 72 °C for 90 seconds, followed by a 72 °C hold for 10 min. PCR2 heated samples to 95 °C for 3 min then comprised 25 cycles of 95 °C for 45 seconds, 55 °C for 60 seconds and 72 °C for 90 seconds, followed by a 72 °C hold for 10 min. Amplicons were pooled and purified using 0.8x NGS beads (NucleoMag), then the library pool was profiled using the Agilent TapeStation prior to sequencing on the MiSeq instrument (2x 250 bp paired-end sequencing).

### Bioinformatic analyses

Demultiplexed forward and reverse FASTQ files for each sample were trimmed and were processed using the DADA2 package in RStudio [58]. Briefly, sequence files were trimmed and underwent quality filtering. Reads were dereplicated, sequences were inferred using the DADA2 algorithm and chimeras were removed. Taxonomy was assigned using the SILVA 138 SSU Ref NR 99 reference database [59]. After removal of control samples, the minimum sample read depth was 9372. Alpha and beta diversity measures, and taxonomic profiles were obtained using the phyloseq package in RStudio [60]. Differential abundance was calculated following TMM (trimmed mean of M values) normalisation with edgeR and with the limma+voom method in RStudio [58, 61, 62]. Briefly, sequence files were trimmed and underwent quality filtering. Reads were dereplicated, sequences were inferred using the DADA2 algorithm and chimeras were removed. Taxonomy was assigned using the SILVA 138 SSU Ref NR 99 reference database [59]. After removal of control samples, the minimum sample read depth was 9372. Alpha and beta diversity measures, and taxonomic profiles were obtained using the phyloseq package in RStudio [60]. Differential abundance was calculated following TMM (trimmed mean of M values) normalisation with edgeR and with the limma+voom method in RStudio [61, 62].

### Statistical analyses

The statistical test used to analyse each dataset is stipulated in the corresponding figure legends. Prism software (version 9; Graph Pad) was used to perform statistical analyses.

## Supplementary information

Supplementary Material text

Figure S1

Figure S2

Figure S3

Supplementary File 1

Reproducibility checklist

## Data Availability

All data and customised Image J macros used by this study are available on request from the authors.

## Materials availability

All materials will be provided under Materials Transfer Agreement upon request to MP (pellegrini@wehi.edu.au).

## References

[1] Brenner D, Blaser H, Mak TW (2015). Regulation of tumour necrosis factor signalling: live or let die. Nat Rev Immunol.

[2] Annibaldi A, Walczak H (2020). Death receptors and their ligands in inflammatory disease and cancer. Cold Spring Harb Perspect Biol.

[3] Holler N, Zaru R, Micheau O, Thome M, Attinger A, Valitutti S (2000). Fas triggers an alternative, caspase-8-independent cell death pathway using the kinase RIP as effector molecule. Nat Immunol.

[4] Dondelinger Y, Delanghe T, Rojas-Rivera D, Priem D, Delvaeye T, Bruggeman I (2017). MK2 phosphorylation of RIPK1 regulates TNF-mediated cell death. Nat Cell Biol.

[5] Annibaldi A, Wicky John S, Vanden Berghe T, Swatek KN, Ruan J, Liccardi G (2018). Ubiquitin-mediated regulation of RIPK1 kinase activity independent of IKK and MK2. Mol Cell.

[6] Jaco I, Annibaldi A, Lalaoui N, Wilson R, Tenev T, Laurien L (2017). MK2 phosphorylates RIPK1 to prevent TNF-induced cell death. Mol Cell.

[7] Gentle IE, Wong WW, Evans JM, Bankovacki A, Cook WD, Khan NR (2011). In TNF-stimulated cells, RIPK1 promotes cell survival by stabilizing TRAF2 and cIAP1, which limits induction of non-canonical NF-kappaB and activation of caspase-8. J Biol Chem.

[8] Wang L, Du F, Wang X (2008). TNF-alpha induces two distinct caspase-8 activation pathways. Cell.

[9] Vince JE, Wong WW, Khan N, Feltham R, Chau D, Ahmed AU (2007). IAP antagonists target cIAP1 to induce TNFalpha-dependent apoptosis. Cell.

[10] Dillon CP, Oberst A, Weinlich R, Janke LJ, Kang TB, Ben-Moshe T (2012). Survival function of the FADD-CASPASE-8-cFLIP(L) complex. Cell Rep.

[11] Micheau O, Tschopp J (2003). Induction of TNF receptor I-mediated apoptosis via two sequential signaling complexes. Cell.

[12] He S, Wang L, Miao L, Wang T, Du F, Zhao L (2009). Receptor interacting protein kinase-3 determines cellular necrotic response to TNF-alpha. Cell.

[13] Cho YS, Challa S, Moquin D, Genga R, Ray TD, Guildford M (2009). Phosphorylation-driven assembly of the RIP1-RIP3 complex regulates programmed necrosis and virus-induced inflammation. Cell.

[14] Sun L, Wang H, Wang Z, He S, Chen S, Liao D (2012). Mixed lineage kinase domain-like protein mediates necrosis signaling downstream of RIP3 kinase. Cell.

[15] Zhao J, Jitkaew S, Cai Z, Choksi S, Li Q, Luo J (2012). Mixed lineage kinase domain-like is a key receptor interacting protein 3 downstream component of TNF-induced necrosis. Proc Natl Acad Sci USA.

[16] Pierdomenico M, Negroni A, Stronati L, Vitali R, Prete E, Bertin J (2014). Necroptosis is active in children with inflammatory bowel disease and contributes to heighten intestinal inflammation. Am J Gastroenterol.

[17] Negroni A, Colantoni E, Pierdomenico M, Palone F, Costanzo M, Oliva S (2017). RIP3 AND pMLKL promote necroptosis-induced inflammation and alter membrane permeability in intestinal epithelial cells. Dig Liver Dis.

[18] Patankar JV, Muller TM, Kantham S, Acera MG, Mascia F, Scheibe K (2021). E-type prostanoid receptor 4 drives resolution of intestinal inflammation by blocking epithelial necroptosis. Nat Cell Biol.

[19] Gunther C, Martini E, Wittkopf N, Amann K, Weigmann B, Neumann H (2011). Caspase-8 regulates TNF-alpha-induced epithelial necroptosis and terminal ileitis. Nature.

[20] Varfolomeev EE, Schuchmann M, Luria V, Chiannilkulchai N, Beckmann JS, Mett IL (1998). Targeted disruption of the mouse Caspase 8 gene ablates cell death induction by the TNF receptors, Fas/Apo1, and DR3 and is lethal prenatally. Immunity.

[21] Kang TB, Ben-Moshe T, Varfolomeev EE, Pewzner-Jung Y, Yogev N, Jurewicz A (2004). Caspase-8 serves both apoptotic and nonapoptotic roles. J Immunol.

[22] Kaiser WJ, Upton JW, Long AB, Livingston-Rosanoff D, Daley-Bauer LP, Hakem R (2011). RIP3 mediates the embryonic lethality of caspase-8-deficient mice. Nature.

[23] Oberst A, Dillon CP, Weinlich R, McCormick LL, Fitzgerald P, Pop C (2011). Catalytic activity of the caspase-8-FLIP(L) complex inhibits RIPK3-dependent necrosis. Nature.

[24] Dillon CP, Weinlich R, Rodriguez DA, Cripps JG, Quarato G, Gurung P (2014). RIPK1 blocks early postnatal lethality mediated by caspase-8 and RIPK3. Cell.

[25] Tisch N, Freire-Valls A, Yerbes R, Paredes I, La Porta S, Wang X (2019). Caspase-8 modulates physiological and pathological angiogenesis during retina development. J Clin Invest.

[26] Muzumdar MD, Tasic B, Miyamichi K, Li L, Luo L (2007). A global double-fluorescent Cre reporter mouse. Genesis.

[27] Salmena L, Lemmers B, Hakem A, Matysiak-Zablocki E, Murakami K, Au PY (2003). Essential role for caspase 8 in T-cell homeostasis and T-cell-mediated immunity. Genes Dev.

[28] Newton K, Dixit VM, Kayagaki N (2021). Dying cells fan the flames of inflammation. Science.

[29] Fritsch M, Gunther SD, Schwarzer R, Albert MC, Schorn F, Werthenbach JP (2019). Caspase-8 is the molecular switch for apoptosis, necroptosis and pyroptosis. Nature.

[30] Alvarez-Diaz S, Dillon CP, Lalaoui N, Tanzer MC, Rodriguez DA, Lin A (2016). The pseudokinase MLKL and the kinase RIPK3 have distinct roles in autoimmune disease caused by loss of death-receptor-induced apoptosis. Immunity.

[31] Stolzer I, Dressel A, Chiriac MT, Neurath MF, Gunther C (2021). An IFN-STAT axis augments tissue damage and inflammation in a mouse model of Crohn’s disease. Front Med (Lausanne).

[32] Schwarzer R, Jiao H, Wachsmuth L, Tresch A, Pasparakis M (2020). FADD and Caspase-8 regulate gut homeostasis and inflammation by controlling MLKL- and GSDMD-mediated death of intestinal epithelial cells. Immunity.

[33] Samson AL, Fitzgibbon C, Patel KM, Hildebrand JM, Whitehead LW, Rimes JS (2021). A toolbox for imaging RIPK1, RIPK3, and MLKL in mouse and human cells. Cell Death Differ.

[34] Samson AL, Garnish SE, Hildebrand JM, Murphy JM (2021). Location, location, location: a compartmentalized view of TNF-induced necroptotic signaling. Sci Signal.

[35] Wittkopf N, Gunther C, Martini E, He G, Amann K, He YW (2013). Cellular FLICE-like inhibitory protein secures intestinal epithelial cell survival and immune homeostasis by regulating caspase-8. Gastroenterology.

[36] He S, Liang Y, Shao F, Wang X (2011). Toll-like receptors activate programmed necrosis in macrophages through a receptor-interacting kinase-3-mediated pathway. Proc Natl Acad Sci USA.

[37] Coats SR, Pham TT, Bainbridge BW, Reife RA, Darveau RP (2005). MD-2 mediates the ability of tetra-acylated and penta-acylated lipopolysaccharides to antagonize Escherichia coli lipopolysaccharide at the TLR4 signaling complex. J Immunol.

[38] Golenbock DT, Hampton RY, Qureshi N, Takayama K, Raetz CR (1991). Lipid A-like molecules that antagonize the effects of endotoxins on human monocytes. J Biol Chem.

[39] Ma F, Zhang J, Zhang J, Zhang C (2010). The TLR7 agonists imiquimod and gardiquimod improve DC-based immunotherapy for melanoma in mice. Cell Mol Immunol.

[40] Berg RD (1996). The indigenous gastrointestinal microflora. Trends Microbiol.

[41] Donaldson GP, Lee SM, Mazmanian SK (2016). Gut biogeography of the bacterial microbiota. Nat Rev Microbiol.

[42] Pinaud L, Sansonetti PJ, Phalipon A (2018). Host cell targeting by enteropathogenic bacteria T3SS effectors. Trends Microbiol.

[43] Pearson JS, Giogha C, Muhlen S, Nachbur U, Pham CL, Zhang Y (2017). EspL is a bacterial cysteine protease effector that cleaves RHIM proteins to block necroptosis and inflammation. Nat Microbiol.

[44] Nissim-Eliraz E, Nir E, Shoval I, Marsiano N, Nissan I, Shemesh H (2017). Type three secretion system-dependent microvascular thrombosis and ischemic enteritis in human gut xenografts infected with enteropathogenic Escherichia coli. Infect Immun.

[45] Duvallet C, Gibbons SM, Gurry T, Irizarry RA, Alm EJ (2017). Meta-analysis of gut microbiome studies identifies disease-specific and shared responses. Nat Commun.

[46] Imhann F, Vich Vila A, Bonder MJ, Fu J, Gevers D, Visschedijk MC (2018). Interplay of host genetics and gut microbiota underlying the onset and clinical presentation of inflammatory bowel disease. Gut.

[47] Kowalska-Duplaga K, Gosiewski T, Kapusta P, Sroka-Oleksiak A, Wedrychowicz A, Pieczarkowski S (2019). Differences in the intestinal microbiome of healthy children and patients with newly diagnosed Crohn’s disease. Sci Rep.

[48] Lehle AS, Farin HF, Marquardt B, Michels BE, Magg T, Li Y (2019). Intestinal inflammation and dysregulated immunity in patients with inherited Caspase-8 deficiency. Gastroenterology.

[49] Ensari A, Marsh MN (2018). Exploring the villus. Gastroenterol Hepatol Bed Bench.

[50] Tisch N, Mogler C, Stojanovic A, Luck R, Korhonen EA, Ellerkmann A (2022). Caspase-8 in endothelial cells maintains gut homeostasis and prevents small bowel inflammation in mice. EMBO Mol Med.

[51] Petrie EJ, Sandow JJ, Lehmann WIL, Liang LY, Coursier D, Young SN (2019). Viral MLKL homologs subvert necroptotic cell death by sequestering cellular RIPK3. Cell Rep.

[52] Wang Y, Nakayama M, Pitulescu ME, Schmidt TS, Bochenek ML, Sakakibara A (2010). Ephrin-B2 controls VEGF-induced angiogenesis and lymphangiogenesis. Nature.

[53] Murphy JM, Czabotar PE, Hildebrand JM, Lucet IS, Zhang JG, Alvarez-Diaz S (2013). The pseudokinase MLKL mediates necroptosis via a molecular switch mechanism. Immunity.

[54] Korner H, Cook M, Riminton DS, Lemckert FA, Hoek RM, Ledermann B (1997). Distinct roles for lymphotoxin-alpha and tumor necrosis factor in organogenesis and spatial organization of lymphoid tissue. Eur J Immunol.

[55] Mombaerts P, Iacomini J, Johnson RS, Herrup K, Tonegawa S, Papaioannou VE (1992). RAG-1-deficient mice have no mature B and T lymphocytes. Cell.

[56] Couter CJ, Surana NK (2016). Isolation and flow cytometric characterization of murine small intestinal lymphocytes. J Vis Exp.

[57] Schindelin J, Arganda-Carreras I, Frise E, Kaynig V, Longair M, Pietzsch T (2012). Fiji: an open-source platform for biological-image analysis. Nat Methods.

[58] Callahan BJ, McMurdie PJ, Rosen MJ, Han AW, Johnson AJ, Holmes SP (2016). DADA2: High-resolution sample inference from Illumina amplicon data. Nat Methods.

[59] Quast C, Pruesse E, Yilmaz P, Gerken J, Schweer T, Yarza P (2013). The SILVA ribosomal RNA gene database project: improved data processing and web-based tools. Nucleic Acids Res.

[60] McMurdie PJ, Holmes S (2013). phyloseq: an R package for reproducible interactive analysis and graphics of microbiome census data. PLoS One.

[61] Law CW, Chen Y, Shi W, Smyth GK (2014). voom: Precision weights unlock linear model analysis tools for RNA-seq read counts. Genome Biol.

[62] Robinson MD, McCarthy DJ, Smyth GK (2010). edgeR: a Bioconductor package for differential expression analysis of digital gene expression data. Bioinformatics.

