# Supplementary material for: Endothelial Caspase-8 prevents fatal necroptotic hemorrhage caused by commensal bacteria
Source: Cell Death Differ. 2022 Jul 23;30(1):27–36. doi: 10.1038/s41418-022-01042-8 (PMC9883523; doi:10.1038/s41418-022-01042-8)
Supplement: Supplementary file 1 — Supplementary Material text [file 41418_2022_1042_MOESM1_ESM.docx]

SUPPLEMENTARY INFORMATION

Endothelial Caspase-8 prevents fatal necroptotic hemorrhage caused by commensal bacteria

Stefanie M. Bader^1,2#^, Simon P. Preston^1,2#^, Katie Saliba^1,2^, Adam Lipszyc^1,2^, Zoe L. Grant^1,2,3^, Liana Mackiewicz^1^, Andrew Baldi^1,2^, Anne Hempel^1^, Michelle P. Clark^1,2^, Thanushi Peiris^1,2^, William Clow^1,2^, Jan Bjelic^1,2^, Michael D Stutz^1,2^, Philip Arandjelovic^1,2^, Jack Teale^1^, Fashuo Du^1,2^, Leigh Coultas^1^, James M. Murphy^1,2^, Cody C Allison^1,2†^, Marc Pellegrini^1,2^*^†^ & Andre L. Samson^1,2^*^†^

^1^ The Walter and Eliza Hall Institute of Medical Research, Melbourne, Australia.

^2^ University of Melbourne, Department of Medical Biology, Melbourne, Australia.

^3^ Gladstone Institutes, San Francisco, CA, USA.

^*^Please address correspondence to: [pellegrini@wehi.edu.au](mailto:pellegrini@wehi.edu.au) (MP) and/or samson.a@wehi.edu.au (ALS)

**Fig. S1. Pathology is restricted to the small intestine despite widespread deletion of endothelial Caspase-8. A** Representative H&E-stained sections of organs from control (C8-flox) and C8-endo mice 14 days after starting tamoxifen dosing. Scale bar indicates 100μM. **B** Fluorescence micrographs of EGFP (green) and tdTomato (magenta) expression in C8-endo-ROSA and control (C8-flox-ROSA) mice. Scale bar indicates 150μM. Data are representative of at least 4 mice per group. **C** H&E-stained sections of skin and peritoneum from LCMV-infected control (C8-flox) and C8-endo mice. Arrows indicate areas of exaggerated non-hemorrhagic edema and leukocyte infiltration. Data are representative of n=9 mice.

**Fig. S2. Unlike other models of intestinal necroptosis, the levels of RIPK3, MLKL and Goblet cells remain unchanged in C8-endo mice. A** Western blot of lysates of the *Ripk3^ko^ Mlkl^ko^* spleen, control (wt) ileum and C8-endo ileum 12 days after starting tamoxifen treatment. Arrowheads indicate full-length forms of MLKL, RIPK3, Caspase-8 and GAPDH. Data are representative of n=3 mice per group. **B** Maximum projection micrographs of the small intestine from the stipulated mice stained for MLKL (magenta) and nuclei (blue). White arrowheads indicate consistent and high expression in the capillaries of control (C8-flox) and C8-endo mice. Data are representative of n=3-5 mice per group. **C** PAS-stained sections of the indicated mice. Arrowheads indicate Goblet cells. Note the absence of Goblet cells in TNF-treated wild-type mice. Data are representative of n=3-10 mice per group.

**Fig. S3. Pathology in C8-endo mice does not rely on TNF from one cell-type, on one bacterial genus or on adaptive immunity.** **A** The proportion of mice that became moribund and required euthanasia. Arrows indicate timing of administration of PBS, an isotype control antibody or anti-TNF antibody. Data are from one cohort (n=5-7 mice per group). * p <0.05 via Log-rank Mantel-cox test. **B** The proportion of chimeric C8-endo mice reconstituted with the indicated bone marrow that became moribund and required euthanasia. Data are from one cohort (n=6-7 mice per group). p = 0.0507 via Log-rank Mantel-cox test. **C** Flow cytometric analyses of cells isolated from the lamina propria of C8-endo mice after administration of an isotype control antibody (n = 20 mice) or antibodies that deplete cells expressing CD3, CD79b or F4/80. The percentage of events with the boxed gates are shown. Data are representative of n=3-7 mice per depletion group. **D** The proportion of control (isotype) or immunodepleted C8-endo mice that became moribund and required euthanasia. Data are from n=3-20 mice per group. ns via Log-rank Mantel-cox test. **E** The proportion of C8-endo chimeric recipient mice reconstituted with the indicated bone marrow that became moribund and required euthanasia. Data are from n=6 mice per group. p = .4540 via Log-rank Mantel-cox test. **F** 16s RNA sequencing was performed on contents of the small intestines from C8-endo mice administered drinking water alone (no ABX; n = 5 mice) or water containing an antibiotic cocktail (ABX; n = 5 mice). Plots show the Alpha diversity of the microbiota in the different experimental groups as expressed by the Richness, Shannon and Simpson indices. * p<0.05, ** p < 0.01 and *** p < 0.001 via pair-wise ANOVA.
