# Supplementary figures and images for: Endothelial Caspase-8 prevents fatal necroptotic hemorrhage caused by commensal bacteria
Source: Cell Death Differ. 2022 Jul 23;30(1):27–36. doi: 10.1038/s41418-022-01042-8 (PMC9883523; doi:10.1038/s41418-022-01042-8)

# Uncropped blots for Fig. S2A

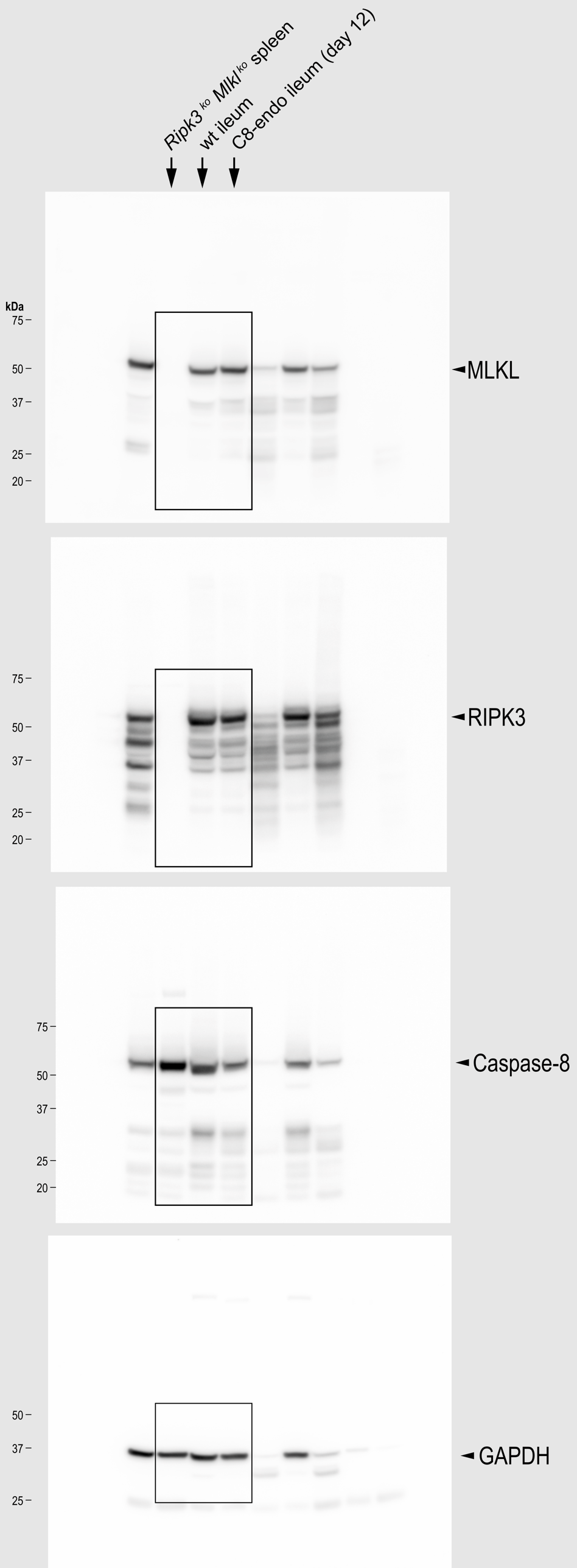

Supplement: Supplementary file 5 — Supplementary File 1 [file 41418_2022_1042_MOESM5_ESM.pdf]
